# Association of Organizational Factors and Physician Practices’ Participation in Alternative Payment Models

**DOI:** 10.1001/jamanetworkopen.2020.2019

**Published:** 2020-04-02

**Authors:** Mariétou H. Ouayogodé, Taressa Fraze, Eugene C. Rich, Carrie H. Colla

**Affiliations:** 1 Department of Population Health Sciences, University of Wisconsin School of Medicine and Public Health, Madison; 2 Department of Family and Community Medicine, School of Medicine, University of California, San Francisco; 3 Mathematica Policy Research, Washington, DC; 4 The Dartmouth Institute for Health Policy & Clinical Practice, Geisel School of Medicine at Dartmouth, Lebanon, New Hampshire

## Abstract

**Importance:**

Consolidation among physician practices and between hospitals and physician practices has accelerated in the past decade, resulting in higher prices in commercial markets. The resulting integration of health care across clinicians and participation in alternative payment models (APMs), which aim to improve quality while constraining spending, are cited as reasons for consolidation, but little is known about the association between integration and APM participation.

**Objective:**

To examine the association of organizational characteristics, ownership, and integration with intensity of participation in APMs among physician practices.

**Design, Setting, and Participants:**

A cross-sectional descriptive study, adjusted for sampling and nonresponse weights, was conducted in US physician practice respondents to the National Survey of Healthcare Organizations and Systems conducted between June 16, 2017, and August 17, 2018; of 2333 responses received (response rate, 46.9%) and after exclusion of ineligible and incomplete responses, the number of practices included in the analysis was 2061. Data analysis was performed from April 1, 2019, to August 31, 2019.

**Exposures:**

Self-reported physician practice characteristics, including ownership, integration (clinical, cultural, financial, and functional), care delivery capabilities, activities, and environmental factors.

**Main Outcomes and Measures:**

Participation in APMs: (1) bundled payments, (2) comprehensive primary care and medical home programs, (3) pay-for-performance programs, (4) capitated contracts with commercial health plans, and (5) accountable care organization contracts.

**Results:**

A total of 49.2% of the 2061 practices included reported participating in 3 or more APMs; most participated in pay-for-performance and accountable care organization models. Covariate-adjusted analyses suggested that operating within a health care system (odds ratio [OR] for medical group: 2.35; 95% CI, 1.70-3.25; *P* < .001; simple health system: 1.46; 95% CI, 1.08-1.97; *P* = .02; and complex health system: 1.76; 95% CI, 1.25-2.47; *P* = .001 relative to independent practices), greater clinical (OR, 4.68; 95% CI, 2.28-9.59; *P* < .001) and functional (OR, 4.24; 95% CI, 2.00-8.97; *P* < .001) integration, and being located in the Northeast (OR for Midwest: 0.47; 95% CI, 0.34-0.65; *P* < .001; South: 0.47; 95% CI, 0.34-0.66; *P* < .001; and West: 0.64; 95% CI, 0.46-0.91; *P* = .01) were associated with greater APM participation.

**Conclusions and Relevance:**

Greater APM participation appears to be supported by integration and system ownership.

## Key Points

**Question:**

Are organizational characteristics, ownership, and integration associated with intensity of participation in alternative payment models among physician practices?

**Findings:**

In this cross-sectional analysis, 2061 physician practice sites in the 2017-2018 National Survey of Healthcare Organizations and Systems were examined. Physician practices operating within health systems and reporting higher levels of clinical and functional integration were more likely to participate in multiple alternative payment models.

**Meaning:**

Health system ownership and greater integration in physician practices appear to be associated with greater alternative payment model participation.

## Teaser

This cross-sectional study examines the association between organizational characteristics, ownership, and integration with intensity of participation in alternative payment models among physician practices.

## Introduction

Since the Patient Protection and Affordable Care Act was passed in 2010, the shift toward value-based payment has promised to reward potential efficiency and quality improvements that may result from integrating care across health care professionals.^1,2^ Despite the expansion of value-based payment models offered by public and private payers, participation in alternative payment models (APMs) may vary^3^ depending on organizational characteristics and structure or geographic area.^4,5,6,7^ There is some evidence that higher levels of integration are associated with a more efficient delivery system^8^ and that integrated medical groups provide better health care quality than independent practices.^9^ Therefore, more integrated health care organizations may be inclined to participate in APMs.

Integration can be classified into 4 subcategories: clinical, financial, cultural, and functional (eFigure 1 in the Supplement).^10,11,12,13,14^ Clinical integration is the extent to which patient care services are coordinated across different functions, activities, and operating units and may strengthen the organization’s ability^15^ to manage care across the continuum.^11^ Clinical integration has been reported to support patient-centered health care, improved continuity of care, and care coordination,^16^ which are often promoted by APMs. Furthermore, successful clinical integration may be facilitated by financial and functional integration.^11,13,17^ Financial integration is the degree of financial management, planning, and control across operating units. Cultural integration refers to relationships and the extent to which knowledge, accountability, common goals, and values are shared across parties involved in the integration.^12,14^ Functional integration involves the exchange of high-quality information to facilitate collaboration systems and technology support through the use of decision and input support tools and organization management information systems.^14^ We hypothesized that each of these types of integration may accelerate adoption of APMs.

Scale remains an important factor in participation; facilities with high patient volumes can better afford high fixed-cost investments and operational changes.^18^ Although economies of scale, which may be facilitated by financial integration, suggest that group practices may improve quality and contain costs via centralized operations,^19^ recent research found that vertical integration (ie, consolidation) is associated with better performance on quality measures but did not significantly reduce use, mortality, spending, or prices.^20^

Prior research demonstrated that accountable care organizations (ACOs) tended to form in locations with better quality, higher Medicare spending, fewer primary care physician groups, greater managed care penetration, less concentrated hospital markets, lower poverty rates, and urban locations.^4,21,22^ Physician practices participating in the Comprehensive Primary Care Plus (CPC+) program were more likely to be located in areas with higher median income, lower poverty rates, higher educational attainment, fewer residents with disability, more commercial insurance, and lower uninsurance rates.^23^ Less is known about factors and organizational characteristics associated with participation in APMs other than ACOs and CPC+ or about overlapping model participation. Practices that participate in multiple APMs may experience greater challenges in aligning quality measurement requirements across payers or APMs.

This study evaluated simultaneous participation of physician practices in APMs, including ACOs, comprehensive primary care and medical homes, pay-for-performance programs, and bundled payments. Our analyses noted characteristics of physician practices more likely to adopt APMs, including innovative survey-based measures of integration, and suggest where additional policy may be needed to increase diffusion.

## Methods

In this cross-sectional study, we examined organizational structure, ownership, care delivery capabilities, integration, and participation in APMs. In-depth information on organizational characteristics, care delivery capabilities, practice activities, and environmental factors were obtained from the National Survey of Healthcare Organizations and Systems (NSHOS), which is a nationally representative survey of primary care and multispecialty physician practices fielded between June 16, 2017, and August 17, 2018. Practices representing single-site locations were identified from the IQVIA OneKey database. OneKey uses information from several sources including proprietary data collection, the American Medical Association’s Physician Masterfile, and publicly available sources to inform on relationships among physicians, practices, hospitals, and health systems. The NSHOS used a stratified-cluster sampling design to select health care systems, physician practices, and hospitals. The sample was stratified based on ownership and composition structures, including samples of both system-owned and independent physician practices and hospitals. Survey weights reflected the sample design and were adjusted for clustering across sampling units and nonresponse.^24^ The NSHOS required physician practices to have at least 3 primary care physicians. A total of 4976 physician practice sites were surveyed and 2333 responses were received, for a response rate of 46.9%. After excluding 143 responses owing to ineligibility^24^ and 129 owing to incomplete responses, the analyzed sample included 2061 practices. Analyses of practices excluded owing to incomplete responses did not suggest significant differences with practices included in this study, although they tended to be smaller (eTable 1 in the Supplement). The institutional review board of Dartmouth College reviewed and approved this study with waiver of informed consent. This study followed the Strengthening the Reporting of Observational Studies in Epidemiology (STROBE) reporting guideline.^25^

### Measures

Intensity of participation in APMs is measured on a scale from 0 to 5 based on whether the practice reported currently participating in (1) bundled or episode-based payments; (2) CPC, CPC+, and patient-centered medical homes; (3) pay-for-performance programs; (4) capitated contracts with commercial health plans; and (5) ACO contracts (Medicare, Medicaid, or commercial).

We included explanatory variables spanning the 4 survey domains: organizational characteristics, care delivery capabilities, practice activities, and environmental factors. Organizational characteristics were measured with binary variables identifying the ownership (ie, health system type) of the practice (independent practice, medical group containing physician practices but no hospitals, simple health system containing practices and hospitals but no owner subsidiary, or complex health system containing practices and hospitals and at least 1 owner subsidiary),^24^ whether the physician practice’s health system includes an academic medical center, size of the practice, and the proportion of primary care physicians in the practice.

We measured payer mix with the percentage of the practice’s revenue from each of the following: commercial health insurance, Medicare, Medicaid, self-pay, or other sources. To measure the perceived competitive environment, we included a dichotomous variable for whether the respondent perceived market competition for patients in the outpatient setting to be intense.

We included measures of 4 types of integration (clinical, functional, cultural, and financial). Clinical, functional, and cultural integration were measured using composite indices calculated as simple weighted sums of components within each integration measure, with equal weight allocated to each component. eTables 2, 3, and 4 in the Supplement present the components included in each measure. Financial integration, defined for practices operating within a larger organization, was measured using a dichotomous variable for whether financial planning and revenue sharing were done systemwide.

In addition, we descriptively reported measures for major barriers to the practices’ use of evidence-based care delivery innovations to gauge practices’ perception about APMs and challenges facing practices participating in APMs. Potential barriers to participation included lack of a process for identifying beneficial innovations, lack of a process for disseminating information about innovations, not having enough time to implement, having insufficient financial resources to implement, lack of the necessary knowledge or expertise to implement, and lack of incentives to implement.

Physician practice zip codes were used to determine urban, suburban (micropolitan), and rural areas.^26^ Practice states were used to identify geographic regions (Northeast, Midwest, South, and West).^27^

### Statistical Analysis

Data analysis was performed from April 1, 2019, to August 31, 2019. In covariate-unadjusted analyses, we compared characteristics and organizational structure of physician practices, categorized by current participation in APMs. We used covariate-adjusted ordered logistic or proportional odds regression models to examine associations between practice-level characteristics, integration and geography measures, and intensity of participation in APMs. The model included practice characteristic and geography measures and clinical, cultural, and functional integration measures. We did not include financial integration in the primary regression model because it was not significantly associated with the likelihood of participation in APMs but substantially reduced the sample of practices included in the analysis. We also tested whether the proportional odds assumption of the ordered logistic model was met. Descriptive covariate-unadjusted characteristics and regression analyses were all adjusted for sampling weights, and SEs were adjusted accordingly.

In sensitivity analyses, we tested whether our main regression model was sensitive to the definition of the outcome variable by conducting separate analyses using logistic regression models for participation in each of the 5 selected APMs. In addition, we estimated generalized linear models that accounted for within-practice connection of participation across models when modeling participation in any APM in relation to covariates included in our main model (practice site characteristics, organizational structure, and market factors). Furthermore, the intensity of participation in APMs may be more related to the fraction of revenues in APMs rather than merely having a contract of any given payment type. Although we were unable to directly measure the fraction of a physician practice’s revenues in each APM, we re-estimated our main regression model by adding an additional covariate—the percentage of the practice’s annual patient care revenues from different payers (eg, commercial health insurance, Medicare, Medicaid, and other) as a proxy—to examine whether participation was connected with these percentages.

Availability of APMs varies across regions based on regulation in some cases and also on the degree of competitiveness of the practice and insurer markets. Recognizing that degree of practice control over participation in APMs might vary by type of model as well as characteristics and location of the practice, we estimated another regression still using the main regression model as a baseline and included dichotomous variables for current availability of patient-centered medical home plans in the state (as of May 2019),^28^ CPC+ eligibility based on Medicare regulation, and measures of insurance market concentration at the hospital referral region level (Medicaid managed care, Medicare Advantage, and private insurance concentration from the 2017 Leavitt Partners market database).^29^ In addition, we estimated the main regression model, stratifying by the practice’s health system type to examine whether the associations estimated differed across system types.

Statistical tests conducted in this study were 2-sided, with *P* < .05 considered significant. Rates of missing data were low except for the measures of financial integration. All statistical analyses were performed with Stata, version 14.2 (StataCorp LLC).

## Results

**Figure 1.**
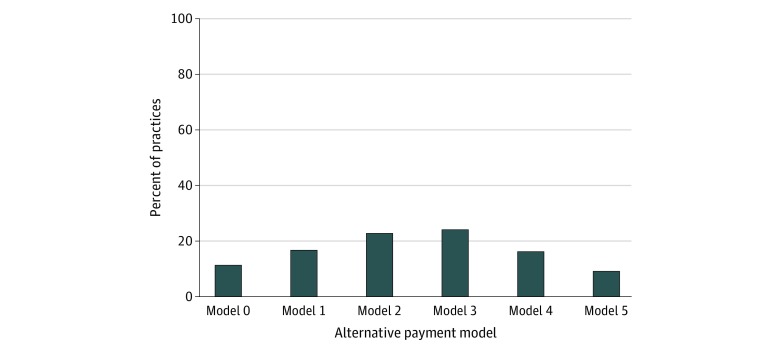
Distribution of Alternative Payment Models Across 2061 Physician Practices Responding to the National Survey of Healthcare Organizations and Systems Alternative payment models included (1) bundled or episode-based payments; (2) comprehensive primary care (CPC), CPC+, and patient-centered medical homes; (3) pay-for-performance programs; (4) capitated contracts with commercial health plans; and (5) accountable care organizations (Medicare, Medicaid, and commercial). Proportions were adjusted for sampling weights.

**Figure 2.**
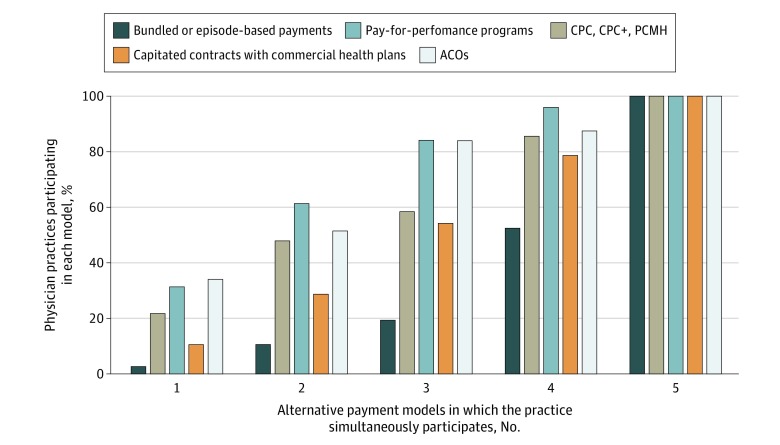
Distribution of Alternative Payment Models (APMs) by Model Type Across 2061 Physician Practices Responding to the National Survey of Healthcare Organizations and Systems Physician Practices Alternative payment models included (1) bundled or episode-based payments; (2) comprehensive primary care (CPC), CPC Plus (CPC+), and patient-centered medical home (PCMH); (3) pay-for-performance programs; (4) capitated contracts with commercial health plans; and (5) accountable care organizations (ACOs) (Medicare, Medicaid, and commercial). Proportions were adjusted for sampling weights. Because the outcome variable measures the number of APMs each practice reported participating in, the only bar that adds up to 100% is the one identifying practices that reported participating in a single APM. When considering the other bars identifying physician practices reporting participation in multiple APMs, the proportions are overlapping and not mutually exclusive; therefore, the sum of proportions in each of these bars exceeds 100%. For example, among physician practices reporting participation in 2 APMs (representing any combination of all 5 selected APMs, 10.6% participate in bundled or episode-based payments (the least common model), 51.5% participate in ACO models, and 61.4% participate in pay-for-performance programs (the 2 most common models). A similar description could be made for physician practices reporting participation in 3, 4, or 5 APMs.

After adjusting for sampling weights accounting for NSHOS complex survey design, 1876 of 2061 physician practices (91.0% [after adjusting for sampling weights]) reported participating in at least 1 APM and 1153 of all practices (49.2%) participated in 3 or more models simultaneously (Figure 1). On a covariate-unadjusted basis, participation in pay-for-performance programs was greatest (31.3% [after adjusting for sampling weights] of 285 practices participating in 1 model, 61.4% of 438 participating in 2 models, 84.1% of 529 participating in 3 models, and 96.0% of 387 participating in 4 models (Figure 2). Accountable care organization models (34.0% [after adjusting for sampling weights] of the 285 practices participating in 1 model, 51.5% of 438 practices participating in 2 models, 83.9% of 529 practices participating in 3 models, and 87.4% of 387 practices participating in 4 models) were also common, whereas bundled payments were less common (2.6% [after adjusting for sampling weights] of those participating in 1 model, 10.6% of those participating in 2 models, 19.4% of those participating in 3 models, and 52.5% of those participating in 4 models) (Figure 2). Considering the distribution of ACO contracts with individual payers (Medicare, Medicaid, and commercial) across the 5 payment model categories, practices reported participating in Medicare ACOs the most, followed by commercial ACOs and Medicaid ACOs (eFigure 2 in the Supplement). When examining practice participation rates by individual alternative payment model category, we still found that pay-for-performance (1384 [64.0% after adjusting for sampling weights]) and ACO models (1375 [60.7%]) were the most common, whereas only 566 practices (25.0%) participated in bundled-payment models (eFigure 3 in the Supplement).

Comparing practice characteristics across participation in APMs showed important differences across groups. Independent practices were less likely to participate in all 5 models compared with those operating in medical groups or complex health systems (31 [16.3%, after adjusting for sampling weights] vs 49 [20.9%] practices operating in medical groups and 127 [50.9%] of those operating in complex health systems). Practices participating in all 5 models were more likely to report including an academic medical center within their health system (98 [37.6%, after adjusting for sampling weights] vs 46 [20.3%] of 185 practices participating in zero initiatives) and have greater than 21 physicians (44 [18.4%] vs 14 [7.4%]) (Table 1).

**Table 1.** Descriptive Statistics of 2061 Physician Practices by Participation in APMs^a^^,^^b^

Variable	Alternative payment models, % (SE)^c^						*P* value^d^
	0	1	2	3	4	5	
**Practice characteristics**							
No. of practices	185	285	438	529	387	237	
System type ^e^^,^^f^							
Independent	56.8 (5.5)	51.3 (4.9)	47.8 (3.9)	38.7 (3.5)	26.6 (3.8)	16.3 (4.1)	<.001
Medical group	7.5 (2.1)	9.4 (1.9)	12.7 (2.0)	15.5 (2.0)	19.9 (2.7)	20.9 (3.3)	<.001
Simple system	9.4 (2.3)	9.1 (1.8)	13.0 (1.9)	12.3 (2.0)	11.3 (2.0)	11.9 (2.6)	.59
Complex system	26.3 (4.9)	30.2 (4.6)	26.5 (3.4)	33.5 (3.5)	42.2 (4.3)	50.9 (5.1)	<.001
Physician practice's health system includes an academic medical center^f^	20.3 (4.5)	22.4 (4.3)	22.7 (3.3)	25.8 (3.6)	32.8 (4.3)	37.6 (5.1)	.03
Practice size^f^							
Small (<10 physicians)	84.1 (3.5)	79.8 (3.3)	77.1 (3.3)	77.1 (2.4)	70.9 (3.2)	67.9 (3.7)	.01
Medium (10-20 physicians)	8.4 (2.4)	8.7 (2.0)	10.8 (2.9)	13.0 (2.0)	12.7 (2.2)	13.7 (2.5)	.40
Large (>20 physicians)	7.4 (2.7)	11.6 (2.7)	12.1 (2.2)	9.9 (1.5)	16.4 (2.4)	18.4 (3.1)	.02
Mean proportion of primary care physicians	64.2 (2.5)	62.5 (2.9)	65.8 (1.8)	66.4 (1.7)	62.9 (1.9)	65.8 (2.0)	.66
Mean % of practice's annual patient care revenue source^g^							
Commercial health insurance	41.0 (2.5)	37.7 (2.3)	38.9 (1.5)	41.6 (1.3)	41.3 (1.6)	42.5 (2.0)	.44
Medicare	33.3 (2.1)	31.4 (2.2)	33.0 (1.4)	32.0 (1.1)	31.3 (1.3)	29.8 (1.5)	.61
Medicaid	13.2 (1.9)	18.1 (2.3)	16.4 (1.4)	16.1 (1.2)	18.8 (1.5)	18.2 (1.8)	.23
Self-pay	7.2 (1.2)	9.6 (2.2)	6.7 (0.8)	6.1 (0.4)	5.0 (0.4)	6.1 (1.0)	.06
Other	5.3 (1.8)	3.1 (0.4)	5.0 (1.0)	4.2 (0.5)	3.6 (0.4)	3.4 (0.4)	.31
Intense market competition for patients in the outpatient setting^f^	71.4 (4.7)	66.6 (4.0)	74.6 (2.8)	71.9 (2.7)	75.4 (3.1)	74.7 (3.7)	.54
Integration variable^h^							
Composite index							
Clinical	0.49 (0.02)	0.52 (0.02)	0.55 (0.01)	0.59 (0.01)	0.65 (0.1)	0.67 (0.1)	<.001
Functional	0.48 (0.03)	0.54 (0.02)	0.55 (0.02)	0.59 (0.01)	0.61 (0.1)	0.66 (0.2)	<.001
Cultural	0.64 (0.02)	0.64 (0.02)	0.63 (0.01)	0.63 (0.01)	0.64 (0.1)	0.64 (0.2)	.96
Financial: systemwide financial planning and revenue sharing ^f^^,^^i^	52.0 (6.1)	44.6 (5.5)	45.5 (4.3)	44.4 (3.4)	48.7 (3.7)	51.4 (5.0)	.76
**Geography**^j^							
Urbanicity^f^							
Urban	75.3 (5.2)	80.8 (3.2)	81.0 (3.2)	86.2 (2.2)	85.5 (2.8)	86.2 (3.0)	.27
Suburban	18.4 (5.2)	9.6 (2.4)	11.3 (3.0)	7.6 (1.8)	8.5 (2.2)	6.3 (2.2)	.29
Rural	6.2 (1.9)	9.6 (2.4)	7.7 (1.8)	86.2 (2.2)	6.0 (1.7)	7.5 (2.2)	.82
Region^f^							
Northeast	11.7 (3.2)	17.4 (3.4)	16.2 (2.6)	29.6 (3.2)	21.6 (3.2)	31.8 (5.0)	<.001
Midwest	24.6 (4.5)	27.9 (4.0)	27.7 (3.4)	20.1 (2.5)	27.9 (3.6)	20.7 (3.4)	.10
South	42.8 (5.5)	32.9 (4.3)	33.3 (3.4)	23.5 (2.7)	25.5 (3.4)	20.2 (3.5)	.002
West	20.9 (5.1)	21.9 (4.8)	22.9 (3.5)	26.8 (3.3)	25.0 (3.6)	27.3 (4.8)	.83

Abbreviations: APMs, alternative payment models; NSHOS, National Survey of Healthcare Organizations and Systems.

^a^ Alternative payment models included (1) bundled or episode-based payments; (2) Comprehensive Primary Care, Comprehensive Primary Care Plus, and patient-centered medical home; (3) pay-for-performance programs; (4) capitated contracts with commercial health plans; and (5) accountable care organizations (Medicare, Medicaid, and commercial).

^b^ All statistics were adjusted for sampling weights.

^c^ Standard errors, which were obtained after accounting for the survey nature of the data in estimating means of each covariate, are reported in lieu of SDs.

^d^ *P* values are reported for testing linear hypotheses for differences in means or proportions across number of alternative payment model categories.

^e^ Medical group contains physician practices but no hospitals, simple system contains practices and hospitals but no owner subsidiary, and complex system contains practices and hospitals and at least 1 owner subsidiary.

^f^ Take the value of 1 if yes, and 0 otherwise.

^g^ Total of 1669.

^h^ Each integration measure composite index (0, 1) is a simple weighted sum of components with equal weight allocated to each component.

^i^ Only practices operating within a larger organization were asked about financial planning and revenue sharing (n = 1413).

^j^ Urban, suburban, and rural areas were defined based on rural-urban commuting area classifications. Urban areas include metropolitan area (core, high commuting, and low commuting). Suburban areas include micropolitan areas (core, high commuting, and low commuting). Rural areas include small towns (core, high commuting, and low commuting), rural areas, and zip code tabulation areas not coded.

Practices participating in all 5 models reported greater clinical integration (composite index score of 0.67 vs 0.49 for practices participating in no models) and functional integration (composite index score of 0.66 vs 0.49 for practices participating in no models). With the exception of financial integration, integration measures were positively correlated with each other. Pairwise correlation analyses showed significant and positive correlation between clinical and functional integration (ρ = 0.4) (eTable 5 in the Supplement), clinical and cultural integration (ρ = 0.2), and functional and cultural integration (ρ = 0.3). Financial integration, where reported, was not correlated with other integration measures.

Compared with practices participating in no APMs, a greater proportion of practices reporting contracts from all models were located in the Northeast (73 [31.8%, after adjusting for sampling weights] vs 21 [11.7%] in practices participating in no models). Lower proportions of these practices were located in the Midwest (59 [20.7%] vs 54 [24.6%]) or South (45 [20.2%] vs 67 [42.8%]). Practices that participated in all models had a higher percentage of annual patient care revenues obtained from commercial health insurance and Medicaid and a lower percentage of revenues obtained from all other sources, but differences across categories were only marginally significant for revenues from self-pay (6.1% vs 7.2% for practices participating in no models) (Table 1).

**Figure 3.**
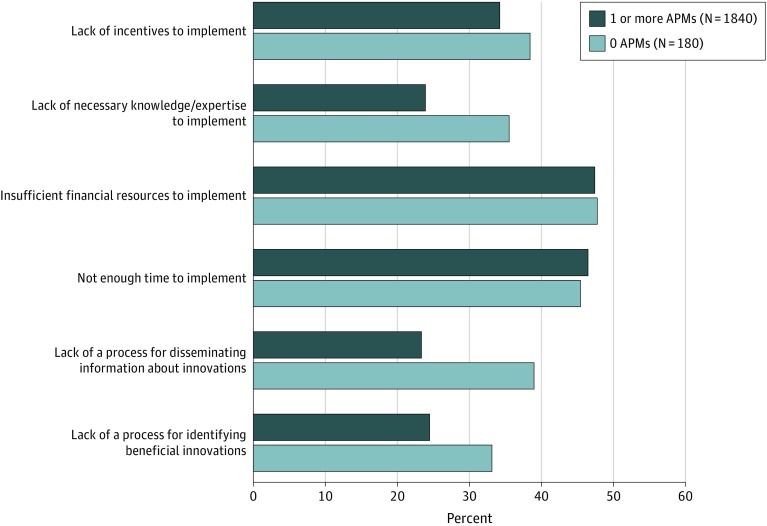
Major Barriers to Use of Evidence-Based Care Delivery Innovations by Participation in Alternative Payment Models Among 2020 Physician Practices The survey respondents were asked to choose among potential barriers to their practice's use of evidence-based care delivery innovations (eg, care transition programs, home visits, or community health workers). For each response option, the respondents specified whether that option constituted a major barrier, a minor barrier, or did not constitute a barrier for their practice.

When reporting major barriers to the practice’s use of evidence-based care delivery innovations, statistically significantly lower proportions of practices participating in APMs indicated barriers, including lack of processes for identifying beneficial innovations (24.4% vs 33.1% of practices participating in no models) (Figure 3), lack of processes for disseminating information about innovations (23.5% vs 38.9%), and lack of the necessary knowledge or expertise to implement innovations (23.9% vs 35.5%). Differences in other reported barriers were not significant at conventional levels.

Covariate-adjusted analysis suggested that operating within a health system (odds ratio [OR] for medical group: 2.35; 95% CI, 1.70-3.25; *P* < .001; simple health system: 1.46; 95% CI, 1.08-1.97; *P* = .02; and complex health system: 1.76; 95% CI, 1.25- 2.47; *P* = .001, relative to independent practices) (Table 2) was significantly associated with a higher likelihood of participation in multiple APMs. Greater clinical (OR, 4.68; 95% CI, 2.28-9.59; *P* < .001) and functional (OR, 4.24; 95% CI, 2.00-8.97; *P* < .001) integration were significantly associated with likelihood of participation in multiple APMs, whereas being located in regions other than the Northeast (OR for Midwest: 0.47; 95% CI, 0.34-0.65; *P* < .001; South: 0.47; 95% CI, 0.34-0.66; *P* < .001; and West: 0.64; 95% CI, 0.46-0.91; *P* = .01) were significantly associated with a lower likelihood. The model did not violate the proportional odds assumption (*F* statistic = 1.10; *P* = .28) (eTable 6 in the Supplement).

**Table 2.** Association Between Physician Practices' Characteristics and Participation in Alternative Payment Models (N = 2061)

Variable	OR (95% CI)	SE	*P* value
**Practice characteristics**			
Practice's health system type^a^			
Independent	1 [Reference]		
Medical group	2.35 (1.70-3.25)	0.39	<.001
Simple system	1.46 (1.08-1.97)	0.22	.02
Complex system	1.76 (1.25- 2.47)	0.30	.001
Practice size^a^			
Small (<10 physicians)	1 [Reference]		
Medium (10-20 physicians)	1.26 (0.91-1.76)	0.21	.16
Large (>20 physicians)	1.17 (0.84-1.62)	0.19	.33
Mean proportion of primary care physicians	1.16 (0.75-1.82)	0.26	.50
Intense market competition for patients in the outpatient setting^a^	1.15 (0.91-1.45)	0.14	.26
Integration^b^			
Composite index (0, 1)			
Clinical	4.68 (2.28-9.59)	1.71	<.001
Functional	4.24 (2.00-8.97)	1.62	<.001
Cultural	0.68 (0.36-1.29)	0.22	.24
**Geography**			
Urbanicity^a^			
Urban	1 [Reference]		
Suburban	0.72 (0.42-1.23)	0.20	.23
Rural	0.89 (0.58-1.37)	0.20	.59
Region^a^			
Northeast	1 [Reference]		
Midwest	0.47 (0.34-0.65)	0.08	<.001
South	0.47 (0.34-0.66)	0.08	<.001
West	0.64 (0.46-0.91)	0.11	.01

Abbreviation: OR, odds ratio.

^a^ Take the value of 1 if yes, and 0 otherwise.

^b^ Each integration measure composite index (0, 1) is a simple weighted sum of components with equal weight allocated to each component. Ordered logit regression models were estimated to capture intensity of participation in alternative payment models, and proportional ORs are reported.

When modeling participation in each of the 5 selected APMs, we found similar results to those in Table 2 with practices operating in larger health systems, practices with greater clinical and functional integration, and those in the Northeast being more likely to participate in individual APMs (eTable 7 in the Supplement). The associations estimated in Table 2 remained robust to the generalized linear models that accounted for participation correlation within practices across APMs (eTable 8 in the Supplement).

When assessing the association between participation in APMs and the proportion of revenues from various payers, we found no statistically significant associations between payer mix and participation in general. However, associations were noted for practices that received more revenue from commercial insurance (OR, 3.04; 95% CI, 0.98-9.46; *P* = .06) (eTable 9 in the Supplement) that were marginally more likely to participate in multiple payment models compared with practices that received more revenues from other sources.

After adjusting for availability of patient-centered medical home plans in the state, CPC+ eligibility based on region, and measures of insurance market concentration at the hospital referral region level, only CPC+ eligibility (OR, 1.32; 95% CI, 1.00-1.74; *P* = .046) (eTable 10 in the Supplement) was significantly associated with a higher likelihood of participation in multiple APMs. The associations estimated in Table 2 remained consistent across practice system ownership types (eTable 11 in the Supplement).

## Discussion

The past decade has seen rapid development and implementation of APMs—federal models and state- and payer-based models—in efforts to bend the health care cost curve and improve quality of care. Although participation in some models is voluntary (eg, ACO, CPC/CPC+), participation in others is mandatory to accelerate adoption of APMs (eg, comprehensive care for joint replacement model) and facilitate evaluation. Given this emphasis on value-based payment,^3^ we used nationally representative survey data to examine organizational and contextual factors associated with physician practices’ participation in APMs. We documented variation in participation across physician practices and observed differences in size, health system type, levels of integration, and geographic location by participation. We found that greater participation in diverse APMs was associated with being in the Northeast, being affiliated with a broader medical group or health care system, and achieving greater clinical and structural integration.

Notwithstanding statistical nonsignificance of some of our estimates, our results align with literature that points to higher participation in models such as ACOs and CPC+ in less concentrated hospital markets, urban locations, and areas with lower poverty rates.^4,21,22,23^ These findings support the idea that practices located in rural areas and areas with higher poverty rates—serving more vulnerable populations—may lack the resources and capabilities to invest in participating in and implementing APMs.^30,31^ Additional incentives may be necessary to encourage health systems and practices operating in rural areas and areas with higher poverty rates to participate in APMs. Research on the ACO investment model, which targets rural and underserved areas, showed that providing up-front financial resources to create the required infrastructure and facilitate participation lowered health care spending and use.^32,33^ Our findings on practice size and system ownership corroborate the idea that scale may reduce random variation in the organization’s performance measures and allow the spreading of fixed costs across a large patient panel.^18^ In addition, relative to smaller health systems and independent practices, larger health systems are exposed to more APMs because of the breadth of services they offer. Moreover, our study augments existing evidence by using unique and rich practice site data to assess the intensity of simultaneous participation in several APMs. Our work also suggests the potential importance of clinical and functional integration in participation in APMs.

### Limitations

Our cross-sectional study has limitations. Health systems are challenging to measure, and practice ownership may be difficult to determine, making interpretation of practice-level data difficult. Nonetheless, the NSHOS addresses this challenge by relying on ownership relationships defined in the IQVIA OneKey database for practices (single-practice site locations). The inclusion criteria for NSHOS restrict surveyed practices to those with greater than 3 primary care physicians, limiting the generalizability of our results to this population. In addition, participation in APMs was self-reported; we are unable to verify the degree of meaningful involvement by physicians.

Our integration measures are not comprehensive. When measuring functional integration, we were unable to measure strategic planning activities that may vary by both organizational size and financial resources. In addition, our cultural integration measures mostly inform on activities happening within the practice rather than on the relationship of the practice with a larger entity. As another limitation, this study provides no information about the performance of practices based on the intensity of participation in APMs, which, although important, is beyond the scope of this study.

## Conclusions

In this study, we found that practice operation within a larger health system appears to be associated with greater APM participation. We also found that participation in APMs seems to be more likely in health systems with markers of greater functional and clinical integration. Under the Medicare Access and Children’s Health Insurance Reauthorization Act of 2015, practices have increasing incentives to participate in various advanced APMs.^34^ Furthermore, the Merit-Based Incentive Payment System may also encourage physician practices to affiliate with larger health care organizations with resources to help physicians succeed and spread the fixed costs of required reporting.^35^ Although greater clinical integration may have the potential to yield higher health care quality or reduced spending, this result is not presently ensured.^36^ Research also suggests the risk of greater administrative costs^19,36^ and higher commercial insurance prices as potential consequences of such consolidation.^37,38^

## Notes

Supplement. **eFigure 1.** Integration Types **eTable 1.** Descriptive Statistics of NSHOS Physician Practices Included (N = 2,061) in Versus Excluded (N = 85) From the Sample Due to Survey Item Non-Response **eTable 2.** Clinical Integration Composite Index Components From the National Survey of Healthcare Organizations and Systems **eTable 3.** Functional Integration Composite Index Components From the National Survey of Healthcare Organizations and Systems **eTable 4.** Cultural Integration Composite Index Components From the National Survey of Healthcare Organizations and Systems **eFigure 2.** Distribution of Alternative Payment Models Across NSHOS Physician Practices by Model Type (N = 2,061) **eFigure 3.** Physician Practice Participation Rates by Individual Alternative Payment Model Category (N = 2,061) **eTable 5.** Matrix of Pair-Wise Correlations Between the Different Types of Integration **eTable 6.** Association Between Physician Practices' Characteristics and Participation in Alternative Payment Models (Test for Proportional Odds Assumption) **eTable 7.** Association Between Physician Practices' Characteristics and Participation in Distinct Alternative Payment Models (Logit Regression Model) **eTable 8.** Association Between Physician Practices' Characteristics and Participation in Alternative Payment Models (Generalized Estimating Equations Regression Model) **eTable 9.** Association Between Physician Practices' Characteristics and Participation in Alternative Payment Models (Controlling for Patient Care Revenue Sources) **eTable 10.** Association Between Physician Practices' Characteristics and Participation in Alternative Payment Models (Controlling for Payment Reform Availability and Market Concentration) **eTable 11.** Association Between Physician Practices' Characteristics and Participation in Distinct Alternative Payment Models Stratified by Practice’s Health System Type
