# Supplementary material for: Association of Organizational Factors and Physician Practices’ Participation in Alternative Payment Models
Source: JAMA Netw Open. 2020 Apr 2;3(4):e202019. doi: 10.1001/jamanetworkopen.2020.2019 (PMC7118519; doi:10.1001/jamanetworkopen.2020.2019)
Supplement: Supplement. — eFigure 1. Integration Types eTable 1. Descriptive Statistics of NSHOS Physician Practices Included (N = 2,061) in Versus Excluded (N = 85) From the Sample Due to Survey Item Non-Response eTable 2. Clinical Integration Composite Index Components From the National Survey of Healthcare Organizations and Systems eTable 3. Functional Integration Composite Index Components From the National Survey of Healthcare Organizations and Systems eTable 4. Cultural Integration Composite Index Components From the National Survey of Healthcare Organizations and Systems eFigure 2. Distribution of Alternative Payment Models Across NSHOS Physician Practices by Model Type (N = 2,061) eFigure 3. Physician Practice Participation Rates by Individual Alternative Payment Model Category (N = 2,061) eTable 5. Matrix of Pair-Wise Correlations Between the Different Types of Integration eTable 6. Association Between Physician Practices' Characteristics and Participation in Alternative Payment Models (Test for Proportional Odds Assumption) eTable 7. Association Between Physician Practices' Characteristics and Participation in Distinct Alternative Payment Models (Logit Regression Model) eTable 8. Association Between Physician Practices' Characteristics and Participation in Alternative Payment Models (Generalized Estimating Equations Regression Model) eTable 9. Association Between Physician Practices' Characteristics and Participation in Alternative Payment Models (Controlling for Patient Care Revenue Sources) eTable 10. Association Between Physician Practices' Characteristics and Participation in Alternative Payment Models (Controlling for Payment Reform Availability and Market Concentration) eTable 11. Association Between Physician Practices' Characteristics and Participation in Distinct Alternative Payment Models Stratified by Practice’s Health System Type [file jamanetwopen-3-e202019-s001.pdf]

Supplementary Online Content

Ouayogodé MH, Fraze T, Rich EC, Colla CH. Association of organizational factors and physician practices' participation in alternative payment models. *JAMA Netw Open*. 2020;3(4):e202019. doi:10.1001/jamanetworkopen.2020.2019

eFigure 1. Integration Types

eTable 1. Descriptive Statistics of NSHOS Physician Practices Included (N=2,061) in Versus Excluded (N=85) From the Sample Due to Survey Item Non-Response

eTable 2. Clinical Integration Composite Index Components From the National Survey of Healthcare Organizations and Systems

eTable 3. Functional Integration Composite Index Components From the National Survey of Healthcare Organizations and Systems

eTable 4. Cultural Integration Composite Index Components From the National Survey of Healthcare Organizations and Systems

eFigure 2. Distribution of Alternative Payment Models Across NSHOS Physician Practices by Model Type (N=2,061)

eFigure 3. Physician Practice Participation Rates by Individual Alternative Payment Model Category (N=2,061)

eTable 5. Matrix of Pair-Wise Correlations Between the Different Types of Integration

eTable 6. Association Between Physician Practices' Characteristics and Participation in Alternative Payment Models (Test for Proportional Odds Assumption)

eTable 7. Association Between Physician Practices' Characteristics and Participation in Distinct Alternative Payment Models (Logit Regression Model)

eTable 8. Association Between Physician Practices' Characteristics and Participation in Alternative Payment Models (Generalized Estimating Equations Regression Model)

eTable 9. Association Between Physician Practices' Characteristics and Participation in Alternative Payment Models (Controlling for Patient Care Revenue Sources)

eTable 10. Association Between Physician Practices' Characteristics and Participation in Alternative Payment Models (Controlling for Payment Reform Availability and Market Concentration)

eTable 11. Association Between Physician Practices' Characteristics and Participation in Distinct Alternative Payment Models Stratified by Practice's Health System Type

This supplementary material has been provided by the authors to give readers additional information about their work.

© 2020 Ouayogodé MH et al. *JAMA Network Open.*

eFigure 1. Integration Types

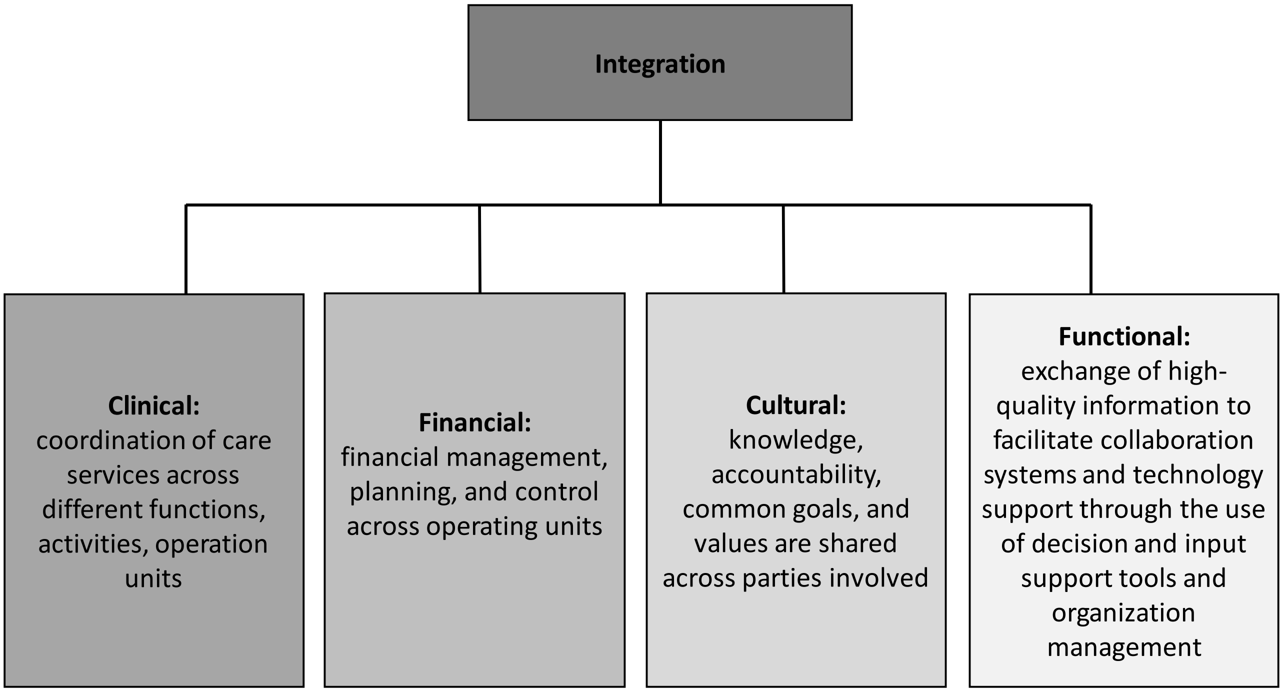

__Sources__: Devers KJ, Shortell SM, Gillies RR, Anderson DA, Mitchell JB, Erickson K. Implementing organized delivery systems: an integration scorecard. *Health Care Management Review.* 1994;19(3):7-20.
Gillies RR, Shortell SM, Anderson DA, Mitchell JB, Morgan KL. Conceptualizing and measuring integration: findings from the health systems integration study. *Journal of Healthcare Management.* 1993;38(4):467.
May C, Finch T, Mair F, et al. Understanding the implementation of complex interventions in health care: the normalization process model. *BMC Health Services Research.* 2007;7(1):148.
Solberg LI, Asche SE, Shortell SM, et al. Is Integration in Large Medical Groups Associated with Quality. *American Journal of Managed Care.* 2009;15(6):e34-e41.
Burns LR, Muller RW. Hospital-Physician Collaboration: Landscape of Economic Integration and Impact on Clinical Integration. *The Milbank Quarterly.* 2008;86(3):375-434.

Note: The figure was created by authors from information compiled from sources listed above.

© 2020 Ouayogodé MH et al. *JAMA Network Open.*

eTable 1. Descriptive statistics of NSHOS physician practices included (N=2,061) in versus excluded (N=85) from the sample due to survey item non-response

**Characteristics**	Mean/Prop	Std. Err.	Mean/Prop	Std. Err.	p-value†
System Type (=1 if yes), % (SE)[a]					
Independent	40.9	(2.4)	49.9	(8.8)	0.3050
Medical group	14.2	(1.3)	16.0	(5.5)	0.7410
Simple system	11.4	(1.1)	6.4	(2.3)	0.0367
Complex system	33.5	(2.9)	27.7	(6.8)	0.3623
Physician practice's health system includes an Academic Medical Center (=1 if yes), % (SE)	26.1	(2.9)	15.5	(5.3)	0.0189
Practice size (=1 if yes), % (SE)[b]					
Small (<10 physicians)	76.5	(1.5)	92.1	(5.9)	0.0094
Medium (10-20 physicians)	11.3	(1.1)	5.2	(5.2)	0.2410
Large (21+ physicians)	12.2	(1.0)	2.7	(2.8)	0.0012
Mean proportion of primary care physicians, % (SE)[c]	64.7	(0.9)	67.9	(7.9)	0.6810
Mean percentage of practice's annual patient care revenues coming from, % (SE)[d]					
Commercial health insurance	40.3	(0.8)	42.6	(5.4)	0.6794
Medicare	31.9	(0.7)	30.5	(3.2)	0.6496
Medicaid	16.8	(0.7)	18.6	(4.0)	0.6535
Self-pay	6.8	(0.5)	6.0	(1.5)	0.6298
Other	4.1	(0.3)	2.3	(0.6)	0.0084
Market competition for patients in the outpatient care intense (=1 if yes), % (SE)[e]	72.4	(1.4)	80.6	(7.4)	0.2720
Major barriers to practice's use of evidence-based care delivery innovations (1=yes), % (SE)[f]					
Lack of a process for identifying beneficial innovations	25.4	(1.4)	24.5	(7.6)	0.9018
Lack of a process for disseminating information about innovations	25.2	(1.4)	21.2	(7.1)	0.5736
Not enough time to implement	46.3	(1.6)	36.0	(8.2)	0.2127
Insufficient financial resources to implement	47.4	(1.6)	38.9	(8.8)	0.3311
Lack the necessary knowledge/expertise to implement	25.2	(1.4)	17.9	(6.6)	0.2746
Lack of incentives to implement	34.7	(1.6)	30.8	(8.3)	0.6454
Integration Variables, mean (SE)[g]					
Clinical: Composite Index {0,1}	0.58	(0.01)	0.52	(0.05)	0.2115
Functional: Composite Index {0,1}	0.57	(0.01)	0.50	(0.04)	0.0835
Cultural: Composite Index {0,1}[h]	0.64	(0.01)	0.60	(0.04)	0.4195
Financial: System wide financial planning and revenue sharing (=1 if yes), % (SE)[i]	46.9	(2.0)	48.5	(10.4)	0.8778
**Geography, % (SE)**					
Urbanicity (=1 if yes)					
Urban	82.8	(1.4)	83.7	(5.9)	0.8794
Suburban	10.0	(1.2)	12.7	(5.3)	0.6249
Rural	7.2	(0.8)	3.7	(2.9)	0.2373
Region (=1 if yes)					
Northeast	21.4	(1.7)	14.4	(6.0)	0.2572
Midwest	24.9	(2.1)	20.9	(5.5)	0.4613
South	29.5	(1.9)	21.8	(6.6)	0.2448
West	24.2	(2.4)	42.9	(9.1)	0.0363
Number of Practices (N)	2061		85		

Abbreviation: NSHOS: National Survey of Healthcare Organizations and Systems; EHR: electronic health records.

Notes: Included (excluded) sample [a] (N=84). [b] (N=21). [c] (N=14), [d] N=1,669(44). [e] (N=63). [f] N=2,020(76). [g] Each integration measure composite index {0,1} is a simple weighted sum of components with equal weight allocated to each component. [h] (N=84). [i] Only practices operating within a larger organization were asked that question, N=1,413(43). Standard errors, which are obtained after accounting for the survey nature of the data in estimating means of each covariate, are reported in parentheses in lieu of standard deviations. Urban, suburban, and rural areas are defined based on rural-urban commuting area classifications. Urban areas include metropolitan area (core, high commuting, low commuting. Suburban areas include micropolitan areas (core, high commuting, low commuting). Rural areas include small towns (core, high commuting, low commuting), rural areas, and ZIP codes tabulation areas not coded. All statistics are adjusted for sampling weights.[†] P-values are reported for testing linear hypotheses for differences in means or proportions between the included practices and excluded practices.

© 2020 Ouayogodé MH et al. *JAMA Network Open.*

eTable 2. Clinical Integration Composite Index Components from the National Survey of Healthcare Organizations and Systems

**Variable**
**q18: How long has your practice had on-site behavioral clinicians?**
**q23: For your complex, high need patients, how often:**
q23a: Is a care manager involved in helping the patient coordinate care across clinicians?
**q24: Which of the following are routinely in place to facilitate complex and high need patients discharge?**
q24a: Referral to community health-related social services (1=Yes)
q24b: Communication with patient within 72 hours of discharge (1=Yes)
q24c: Home visit after discharge (1=Yes)
q24d: Discharge summaries sent to primary care clinician within 72 hours of discharge (1=Yes)
q24e: Standardized process to reconcile multiple medications (1=Yes)
**q37: Does your practice's EHR connect directly to the EHR at the main hospital that your patients use?**

Abbreviation: EHR: electronic health records.

© 2020 Ouayogodé MH et al. *JAMA Network Open.*

eTable 3. Functional Integration Composite Index Components from the National Survey of Healthcare Organizations and Systems

**Variable**
**q6: How often do these things happen within your practice?**
q6h: Our physicians deliver clinical care for patients using the same protocols and pathways
**q36: How often do clinicians in your practice have access to the following when they need it?**
q36a: Notification that a patient was admitted to a local hospital
q36b: Notification that a patient visited an emergency department at a local hospital
q36c: Discharge summaries from local hospitals
q36d: Labs/test results
q36e: Behavioral health notes
q36f: Recommendations and results from specialist consultations
q36g: Information from groups that are not using your EHR
q36h: Information from local, public social service agencies (e.g. county or city shelters, social workers, food programs)
**q48: Does your practice collect information about individual clinician performance for:**
q48a: Diabetes (1=Yes)
q48b: Congestive heart failure (1=Yes)
q48c: Asthma / COPD (1=Yes)
q48d: Coronary artery disease (1=Yes)
q48e: Hypertension (1=Yes)
q48f: Depression (1=Yes)
q48g: Serious mental illness (1=Yes)
**q49: Management of information about individual clinician performance for:**
q49a: Preventive services (e.g. immunizations, screening)
q49a1: Use for feedback
q49a2: Use for internal quality improvement
q49b: Patient experiences (e.g. patient satisfaction or CAHPS scores)
q49b1: Use for feedback
q49b2: Use for internal quality improvement

Abbreviation: EHR: electronic health records; COPD: chronic obstructive pulmonary disease; CAHPS: consumer assessment of healthcare providers and systems; HEDIS: healthcare effectiveness data and information set.

© 2020 Ouayogodé MH et al. *JAMA Network Open.*

eTable 3. Functional Integration Composite Index Components from the National Survey of Healthcare Organizations and Systems *(Continued)*

Variable
**q49: Management of information about individual clinician performance for *(Continued)*:**
q49c: Overuse of medical tests or procedures (e.g. high cost imaging)
q49c1: Use for feedback
q49c2: Use for internal quality improvement
q49d: Underuse of medical test or procedures (e.g. HEDIS)
q49d1: Use for feedback
q49d2: Use for internal quality improvement
q49e: Use of acute care services (e.g. readmissions, emergency room use)
q49e1: Use for feedback
q49e2: Use for internal quality improvement
q49f: Clinical quality (e.g. blood pressure control, diabetes control, complication rates)
q49f1: Use for feedback
q49f2: Use for internal quality improvement
q49g: Total inpatient cost of care
q49g1: Use for feedback
q49g2: Use for internal quality improvement
**q50: Are reports shared within the group in a way that an individual clinician can compare their performance to other clinicians within the practice? (1=Yes)**

Abbreviation: EHR: electronic health records; COPD: chronic obstructive pulmonary disease; CAHPS: consumer assessment of healthcare providers and systems; HEDIS: healthcare effectiveness data and information set.

© 2020 Ouayogodé MH et al. *JAMA Network Open.*

eTable 4. Cultural Integration Composite Index Components from the National Survey of Healthcare Organizations and Systems

Variable
**q6: How often do these things happen within your practice?**
q6a: Successful care delivery innovations are highly publicized within the practice
q6b: Team members openly share patient care challenges and failures with each other
q6c: There is protected time given to generate new ideas and innovations
q6d: We encourage trying new ideas to see if they work
q6e: There is a strong sense of belonging to this practice
q6f: We consider ourselves to be the testing ground for new approaches to engage patients in their care
q6g: Team members feel safe raising concerns regarding patient care
q6i: Non-clinical decisions about the practice are made by our physicians acting as a single integrated group
**q7: Which statement best describes your practice?** *(100 points distributed across response options)*
q7a: Our practice is a very personal place. It is a lot like an extended family. People seem to share a lot of themselves.
q7b: ...is a very dynamic and entrepreneurial place. People are willing to try new things to see if they work.
q7c: ...is a very formalized and structured place. Bureaucratic procedures generally govern what people do.
q7d: ...is very production oriented. The major concern is getting the job done.
**q59: How much influence would you say that physicians in your practice have on the setting of practice priorities and strategies?**

© 2020 Ouayogodé MH et al. *JAMA Network Open.*

eFigure 2. Distribution of alternative payment models across NSHOS physician practices by model type (N=2,061)

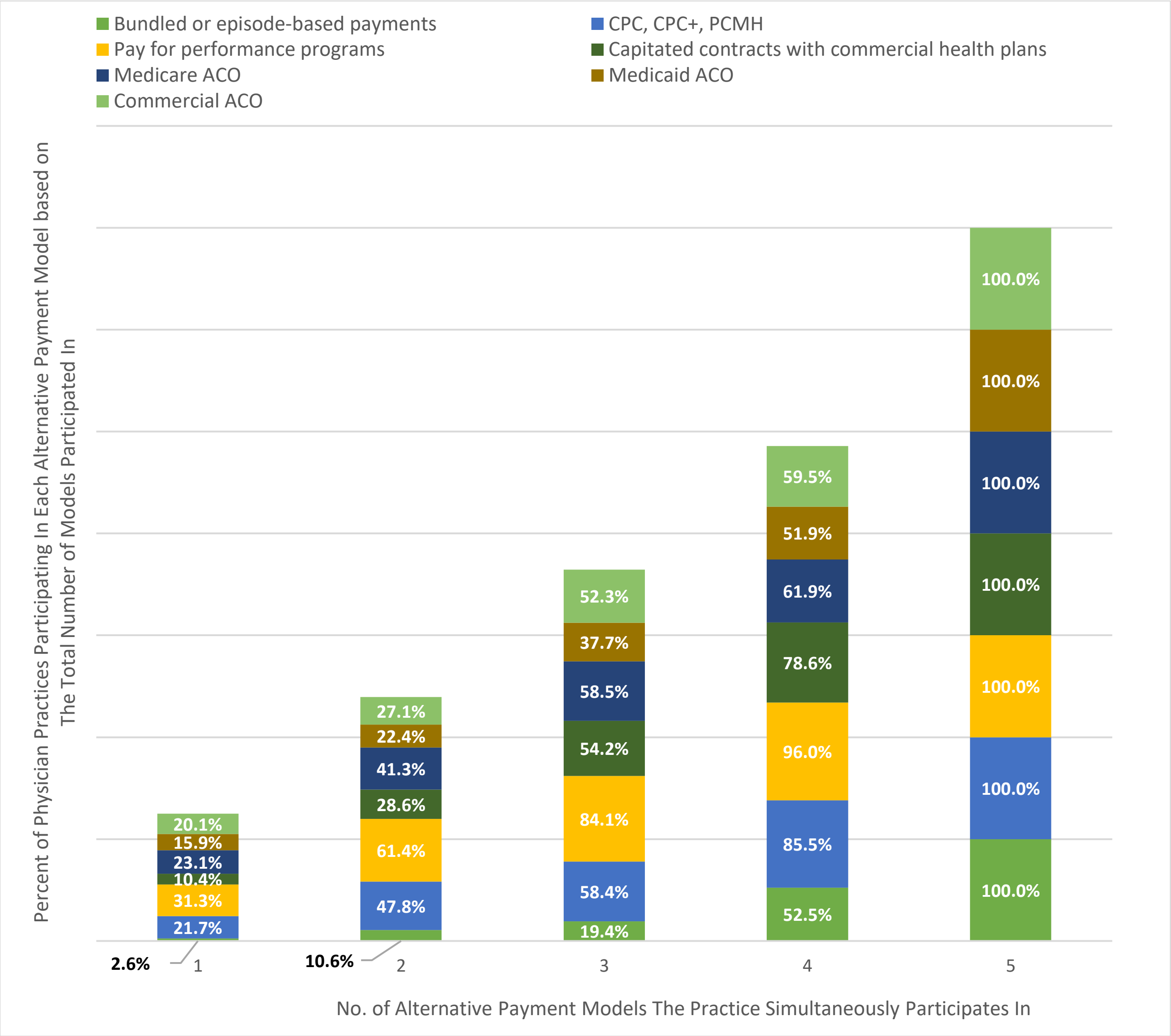

Notes: Abbreviation: NSHOS: National Survey of Healthcare Organizations and Systems. Alternative payment models included a) bundled or episode-based payments, b) comprehensive primary care (CPC), CPC+, patient centered medical homes (PCMH), c) pay for performance programs, d) capitated contracts with commercial health plans, e) accountable care organizations (ACOs) (Medicare, Medicaid, commercial). Proportions are adjusted for sampling weights.

Because the outcome variable measures the number of APMs each practice reported participating in, the only bar that adds up to 100% is the one identifying practices that reported participating into a single APM. When considering the other bars identifying physician practices reporting participation in multiple APMs, the proportions are overlapping and not mutually exclusive, therefore the sum of proportions in each of these bars exceeds 100%. For example, among physician practices reporting participating in 2 APMs (representing any combination of all 5 selected APMs), 10.6% participate in bundled or episode-based payments, nearly half (47.8%) participate in CPC, CPC+, or PCMH, 61.4% participate in pay-for-performance programs, 28.6% participate in capitated contracts with commercial health plans, 41.3% participate in Medicare ACO models, 22.4% participate in Medicaid ACO models, and 27.1% participate in commercial ACO models. A similar description could be made for physician practices reporting participating in 3, 4, or 5 APMs.

© 2020 Ouayogodé MH et al. *JAMA Network Open.*

eFigure 3. Physician practice participation rates by individual alternative payment model category (N=2,061)

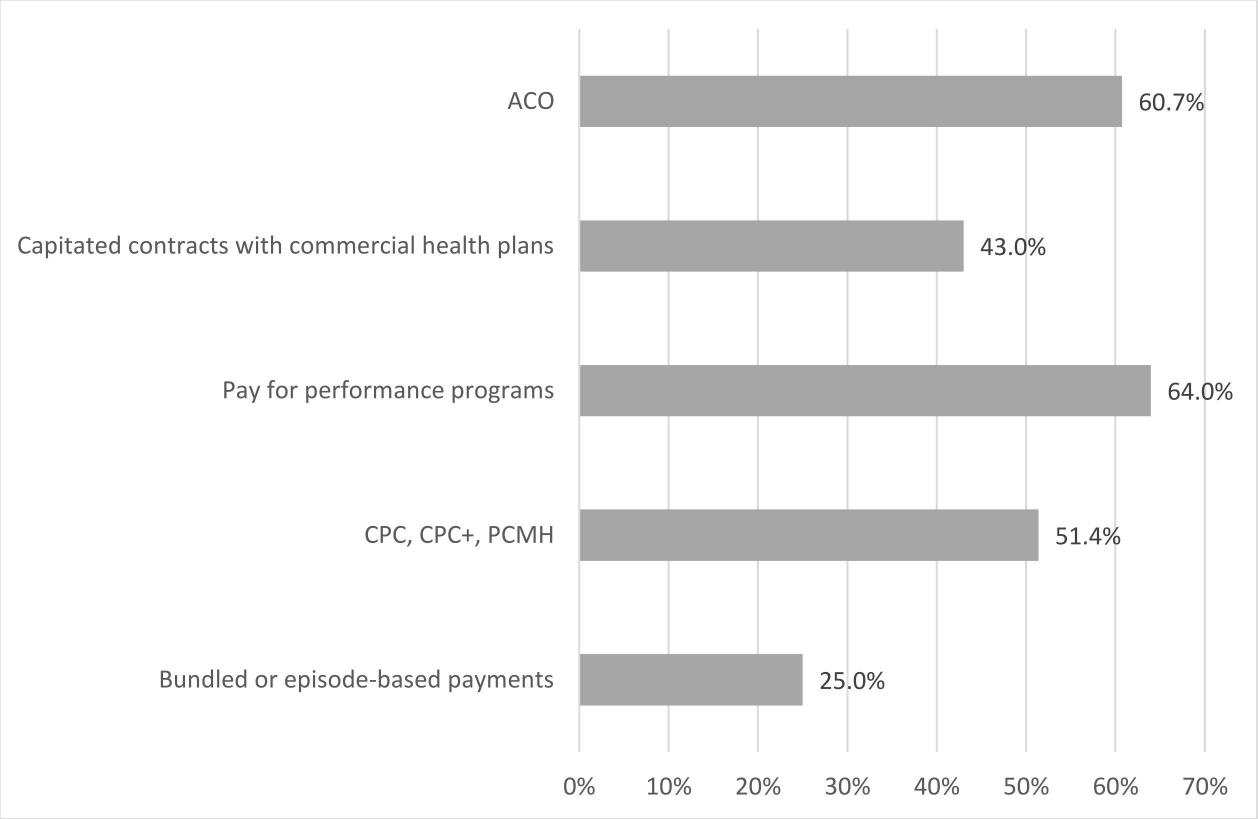

Notes: Alternative payment models included a) bundled or episode-based payments, b) comprehensive primary care (CPC), CPC+, patient centered medical homes (PCMH), c) pay for performance programs, d) capitated contracts with commercial health plans, e) accountable care organizations (ACO) (Medicare, Medicaid, commercial).

© 2020 Ouayogodé MH et al. *JAMA Network Open.*

eTable 5. Matrix of pair-wise correlations between the different types of integration

**Integration Type**	Clinical: Composite Index	Functional: Composite Index	Cultural: Composite Index	Financial: System wide sharing
Clinical: Composite Index {0,1}	1			
Functional: Composite Index {0,1}	0.3765***	1		
Cultural: Composite Index {0,1}	0.1665***	0.2966***	1	
Financial: System wide financial planning and resource sharing (=1 if yes)	0.0279	-0.0116	-0.1264***	1

Notes: Inference: ***p<0.01 after Bonferroni adjustment

© 2020 Ouayogodé MH et al. *JAMA Network Open.*

eTable 6. Association between physician practices' characteristics and participation in alternative payment models (Test for proportion odds assumption)

	(1)	(2)	(3)	(4)	(5)
Variables	Coeff	Coeff	Coeff	Coeff	Coeff
**Practice Characteristics**					
Practice's health system type (=1 if yes)					
Independent *(reference)*					
Medical group	0.92***	0.92***	0.92***	0.92***	0.92***
	(0.18)	(0.18)	(0.18)	(0.18)	(0.18)
Simple system	0.40**	0.40**	0.40**	0.40**	0.40**
	(0.16)	(0.16)	(0.16)	(0.16)	(0.16)
Complex system	0.31	0.27	0.53***	0.79***	0.94***
	(0.24)	(0.21)	(0.18)	(0.19)	(0.24)
Practice size (=1 if yes)					
Small (<10 physicians) *(reference)*					
Medium (10-20 physicians)	0.24	0.24	0.24	0.24	0.24
	(0.17)	(0.17)	(0.17)	(0.17)	(0.17)
Large (21+ physicians)	0.17	0.17	0.17	0.17	0.17
	(0.16)	(0.16)	(0.16)	(0.16)	(0.16)
Mean proportion of primary care physicians	0.15	0.15	0.15	0.15	0.15
	(0.23)	(0.23)	(0.23)	(0.23)	(0.23)
Market competition for patients in the outpatient intense (=1 if yes)	0.14	0.14	0.14	0.14	0.14
	(0.12)	(0.12)	(0.12)	(0.12)	(0.12)
**Integration†**					
Clinical: Composite Index {0,1}	1.55***	1.55***	1.55***	1.55***	1.55***
	(0.37)	(0.37)	(0.37)	(0.37)	(0.37)
Functional: Composite Index {0,1}	1.44***	1.44***	1.44***	1.44***	1.44***
	(0.38)	(0.38)	(0.38)	(0.38)	(0.38)
Cultural: Composite Index {0,1}	-0.40	-0.40	-0.40	-0.40	-0.40
	(0.32)	(0.32)	(0.32)	(0.32)	(0.32)
**Geography**					
Urbanicity (=1 if yes)					
Urban *(reference)*					
Suburban	-0.34	-0.34	-0.34	-0.34	-0.34
	(0.27)	(0.27)	(0.27)	(0.27)	(0.27)
Rural	-0.11	-0.11	-0.11	-0.11	-0.11
	(0.22)	(0.22)	(0.22)	(0.22)	(0.22)
Region (=1 if yes)					
Northeast *(reference)*					
Midwest	-0.76***	-0.76***	-0.76***	-0.76***	-0.76***
	(0.17)	(0.17)	(0.17)	(0.17)	(0.17)
South	-0.77***	-0.77***	-0.77***	-0.77***	-0.77***
	(0.17)	(0.17)	(0.17)	(0.17)	(0.17)
West	-0.46**	-0.46**	-0.46**	-0.46**	-0.46**
	(0.18)	(0.18)	(0.18)	(0.18)	(0.18)
**Wald test of parallel lines assumption**					
F- statistic [p-value]	1.1 [0.2828]				
Observations	2061				

Abbreviation: EHR: electronic health record. Notes: †Each integration measure composite index {0,1} is a simple weighted sum of components with equal weight allocated to each component. Each column presents results from the regression at each of the (6-1=5; {0,1,2,3,4}) values taken by the dependent variable (participation in alternative payment models={0,1,2,3,4,5}). Generalized ordered logit regression model, to do a global test of the proportional odds/parallel linear assumption, was estimated. The model also tested whether a partial proportional odds model could fit the data and regression coefficients (coeff) were reported. Standard errors are in parentheses. Inference: * p<0.10, ** p<0.05, *** p<0.01

© 2020 Ouayogodé MH et al. *JAMA Network Open.*

eTable 7. Association between physician practices' characteristics and participation in distinct alternative payment models (Logit regression model)

	(1)	(2)	(3)	(4)	(5)
Variables	Odds Ratio	Odds Ratio	Odds Ratio	Odds Ratio	Odds Ratio
**Practice Characteristics**					
Practice's health system type (=1 if yes)					
Independent *(reference)*					
Medical group	2.06***	2.39***	1.71***	1.68***	1.77***
	(0.45)	(0.53)	(0.34)	(0.32)	(0.36)
Simple system	1.44	1.65**	1.16	1.34	1.27
	(0.33)	(0.34)	(0.24)	(0.26)	(0.26)
Complex system	1.87***	1.38*	1.30	1.52**	1.61**
	(0.39)	(0.26)	(0.26)	(0.27)	(0.33)
Practice size (=1 if yes)					
Small (<10 physicians) *(reference)*					
Medium (10-20 physicians)	1.40	1.30	0.94	0.96	1.31
	(0.37)	(0.33)	(0.20)	(0.19)	(0.30)
Large (21+ physicians)	1.51***	1.26	1.07	1.06	0.80
	(0.26)	(0.25)	(0.22)	(0.21)	(0.16)
Mean proportion of primary care physicians	0.78	0.85	2.13***	1.48	0.77
	(0.23)	(0.23)	(0.60)	(0.39)	(0.22)
Market competition for patients in the outpatient intense (=1 if yes)	1.22	1.12	0.96	1.01	1.16
	(0.20)	(0.16)	(0.14)	(0.14)	(0.17)
**Integration†**					
Clinical: Composite Index {0,1}	3.50**	6.52***	2.12*	1.92*	2.36**
	(1.56)	(2.52)	(0.90)	(0.74)	(0.96)
Functional: Composite Index {0,1}	2.54*	6.52***	2.72**	1.01	4.27***
	(1.32)	(2.90)	(0.15)	(0.38)	(1.74)
Cultural: Composite Index {0,1}	0.77	0.48*	0.75	1.00	1.02
	(0.31)	(0.18)	(0.29)	(0.33)	(0.37)
**Geography**					
Urbanicity (=1 if yes)					
Urban *(reference)*					
Suburban	1.67	0.97	0.67	0.53**	0.75
	(0.54)	(0.30)	(0.17)	(0.14)	(0.20)
Rural	1.28	1.35	1.55	0.61*	0.56**
	(0.33)	(0.36)	(0.43)	(0.15)	(0.14)
Region (=1 if yes)					
Northeast *(reference)*					
Midwest	0.63**	0.49***	0.50***	0.70**	0.56***
	(0.13)	(0.10)	(0.10)	(0.13)	(0.11)
South	0.76	0.50***	0.56***	0.60**	0.52***
	(0.16)	(0.10)	(0.11)	(0.12)	(0.11)
West	0.91	0.54***	0.70	1.15	0.49***
	(0.21)	(0.12)	(0.16)	(0.23)	(0.11)
Observations	2061	2061	2061	2061	2061

Abbreviation: EHR: electronic health record.

Notes: †Each integration measure composite index {0,1} is a simple weighted sum of components with equal weight allocated to each component. Each column presents results from separate regressions: (1) Bundled or episode-based payments ; (2) CPC, CPC+, PCMH; (3) Pay for performance programs; (4) Capitated contracts with commercial health plans; (5) ACO. Logit regression models, to capture participation in individual alternative payment models, were estimated and odds ratios were reported. Standard errors are in parentheses. Inference: * p<0.10, ** p<0.05, *** p<0.01

© 2020 Ouayogodé MH et al. *JAMA Network Open.*

eTable 8. Association between physician practices' characteristics and participation in alternative payment models (Generalized Estimating Equations regression model)

Variables	Odds Ratio	Std. Err.	
Bundled or episode-based payments *(reference)*			
CPC, CPC+, PCMH	3.40	(0.28)	***
Pay for performance programs	5.90	(0.55)	***
Capitated contracts with commercial health plans	2.37	(0.20)	***
ACO (Medicare, Medicaid or Commercial)	5.10	(0.49)	***
**Practice Characteristics**			
Practice's health system type (=1 if yes)			
Independent *(reference)*			
Medical group	1.86	(0.20)	***
Simple system	1.34	(0.15)	***
Complex system	1.49	(0.15)	***
Practice size (=1 if yes)			
Small (<10 physicians) *(reference)*			
Medium (10-20 physicians)	1.13	(0.12)	
Large (21+ physicians)	1.10	(0.13)	
Mean proportion of primary care physicians	1.13	(0.17)	
Market competition for patients in the outpatient intense (=1 if yes)	1.07	(0.09)	
**Integration†**			
Clinical: Composite Index {0,1}	2.88	(0.65)	***
Functional: Composite Index {0,1}	2.88	(0.67)	***
Cultural: Composite Index {0,1}	0.77	(0.16)	
**Geography**			
Urbanicity (=1 if yes)			
Urban *(reference)*			
Suburban	0.78	(0.13)	
Rural	0.95	(0.14)	
Region (=1 if yes)			
Northeast *(reference)*			
Midwest	0.58	(0.06)	***
South	0.59	(0.06)	***
West	0.73	(0.08)	***
Observations	10305		

Abbreviation: EHR: electronic health record.
Notes: †Each integration measure composite index {0,1} is a simple weighted sum of components with equal weight allocated to each component. A generalized estimating equations (GEE) regression model with binomial distribution of outcome variable was estimated and odds ratios were reported. The dataset was re-arranged as a panel allowing each practice to have five different options/observations based on five types of alternative payment models studied, hence the increase in the number of observations (N=2061*5=10305). The GEE model fits a generalized linear model that adjusts for within-practice correlation of participation in alternative payment models. The dependent variable is a dichotomous variable for participation in any alternative payment model. The model also estimates alternative payment model fixed effects (bundled payments is the reference) Inference: * p<0.10, ** p<0.05, *** p<0.01

eTable 9. Association between physician practices' characteristics and participation in alternative payment models (Controlling for patient care revenue sources)

Variables	Odds Ratio	Std. Err.	
**Practice Characteristics**			
Practice's health system type (=1 if yes)			
Independent *(reference)*			
Medical group	2.29	(0.41)	***
Simple system	1.60	(0.28)	***
Complex system	1.86	(0.34)	***
Practice size (=1 if yes)			
Small (<10 physicians) *(reference)*			
Medium (10-20 physicians)	1.22	(0.23)	
Large (21+ physicians)	1.03	(0.21)	
Mean proportion of primary care physicians	1.11	(0.29)	
Market competition for patients in the outpatient intense (=1 if yes)	1.14	(0.15)	
**Integration†**			
Clinical: Composite Index {0,1}	5.72	(2.39)	***
Functional: Composite Index {0,1}	4.13	(1.76)	***
Cultural: Composite Index {0,1}	0.68	(0.25)	
Mean percentage of practice's annual patient care revenue sources			
Commercial health insurance	3.04	(1.76)	*
Medicare	1.38	(0.83)	
Medicaid	2.64	(1.79)	
Other *(reference)*			
**Geography**			
Urbanicity (=1 if yes)			
Urban *(reference)*			
Suburban	0.72	(0.23)	
Rural	0.85	(0.22)	
Region (=1 if yes)			
Northeast *(reference)*			
Midwest	0.46	(0.08)	***
South	0.55	(0.10)	***
West	0.67	(0.13)	**
Observations	1669		

Abbreviation: EHR: electronic health record.
Notes: †Each integration measure composite index {0,1} is a simple weighted sum of components with equal weight allocated to each component. An ordered logit regression model, to capture intensity of participation in alternative payment models, was estimated and proportional odds ratios were reported. Inference: * p<0.10, ** p<0.05, *** p<0.01

© 2020 Ouayogodé MH et al. *JAMA Network Open.*

eTable 10. Association between physician practices' characteristics and participation in alternative payment models (Controlling for payment reform availability and market concentration)

Variables	Odds Ratio	Std. Err.	
**Practice Characteristics**			
Practice's health system type (=1 if yes)			
Independent *(reference)*			
Medical group	2.43	(0.40)	***
Simple system	1.47	(0.23)	**
Complex system	1.81	(0.31)	***
Practice size (=1 if yes)			
Small (<10 physicians) *(reference)*			
Medium (10-20 physicians)	1.30	(0.22)	
Large (21+ physicians)	1.19	(0.20)	
Mean proportion of primary care physicians	1.11	(0.25)	
Market competition for patients in the outpatient intense (=1 if yes)	1.17	(0.14)	
Payment reform availability and market concentration			
PCMH health plan sold in state (=1 if yes)	1.20	(0.22)	
CPC+ eligible region (=1 if yes)	1.32	(0.19)	**
HHI at the HRR level: Medicaid managed care	1.50	(1.10)	
HHI at the HRR level: Medicare advantage	2.38	(1.32)	
HHI at the HRR level: Private insurance	0.62	(0.19)	
**Integration†**			
Clinical: Composite Index {0,1}	4.48	(1.66)	***
Functional: Composite Index {0,1}	4.11	(1.58)	***
Cultural: Composite Index {0,1}	0.69	(0.22)	
**Geography**			
Urbanicity (=1 if yes)			
Urban *(reference)*			
Suburban	0.69	(0.18)	
Rural	0.86	(0.20)	
Region (=1 if yes)			
Northeast *(reference)*			
Midwest	0.42	(0.07)	***
South	0.41	(0.07)	***
West	0.59	(0.12)	**
Observations	2061		

Abbreviations: EHR: electronic health record; PCMH: patient-centered medical home; CPC: comprehensive primary care; HHI: Herfindahl-Hirschman Index; HRR: Hospital referral region.
Notes: †Each integration measure composite index {0,1} is a simple weighted sum of components with equal weight allocated to each component. An ordered logit regression model, to capture intensity of participation in alternative payment models, was estimated and proportional odds ratios were reported. Inference: * p<0.10, ** p<0.05, *** p<0.01

© 2020 Ouayogodé MH et al. *JAMA Network Open.*

eTable 11. Association between physician practices' characteristics and participation in distinct alternative payment models stratified by practice's health system type

	Practice's Health System Type			
	Independent	Medical Group	Simple System	Complex system
Variables	Odds Ratio	Odds Ratio	Odds Ratio	Odds Ratio
**Practice Characteristics**				
Practice size (=1 if yes)				
Small (<10 physicians) *(reference)*				
Medium (10-20 physicians)	1.83	0.80	0.80	1.05
	(0.67)	(0.24)	(0.27)	(0.25)
Large (21+ physicians)	1.67	1.30	1.46	0.93
	(0.70)	(0.36)	(0.43)	(0.23)
Mean proportion of primary care physicians	0.73	1.88	1.67	1.38
	(0.32)	(0.84)	(0.74)	(0.43)
Market competition for patients in the outpatient intense (=1 if yes)	1.09	1.19	1.11	1.12
	(0.26)	(0.29)	(0.30)	(0.17)
**Integration†**				
Clinical: Composite Index {0,1}	10.74***	1.06	3.20*	2.77*
	(7.05)	(0.76)	(2.09)	(1.68)
Functional: Composite Index {0,1}	1.81	8.63***	26.40***	6.42***
	(1.19)	(6.33)	(20.02)	(3.93)
Cultural: Composite Index {0,1}	0.72	0.80	0.15	1.14
	(0.43)	(0.53)	(0.11)	(0.58)
**Geography**				
Urbanicity (=1 if yes)				
Urban *(reference)*				
Suburban	0.54	0.78	0.72	1.07
	(0.25)	(0.52)	(0.26)	(0.35)
Rural	1.19	0.50	0.99	0.65
	(0.52)	(0.27)	(0.32)	(0.21)
Region (=1 if yes)				
Northeast *(reference)*				
Midwest	0.37***	1.12	0.60*	0.40***
	(0.12)	(0.47)	(0.17)	(0.11)
South	0.43***	0.84	0.32***	0.40***
	(0.13)	(0.34)	(0.10)	(0.13)
West	0.51**	1.06	0.64	0.66
	(0.17)	(0.36)	(0.23)	(0.21)
Observations	586	360	308	807

Abbreviation: EHR: electronic health record.

Notes: †Each integration measure composite index {0,1} is a simple weighted sum of components with equal weight allocated to each component. Each column presents results from separate regressions. Ordered logit regression models, to capture intensity of participation in alternative payment models, were estimated and proportional odds ratios were reported. Standard errors are in parentheses. Inference: * p<0.10, ** p<0.05, *** p<0.01

© 2020 Ouayogodé MH et al. *JAMA Network Open.*
